# Impacts of Phenolic Compounds and Their Benefits on Human Health: Germination

**DOI:** 10.3390/metabo15070425

**Published:** 2025-06-22

**Authors:** Jonathan Hernández-Miranda, Karen Argelia Reyes-Portillo, Abigail García-Castro, Esther Ramírez-Moreno, Alma Delia Román-Gutiérrez

**Affiliations:** 1 Área Académica de Química, Instituto de Ciencias Básicas e Ingeniería, Universidad Autónoma del Estado de Hidalgo, Carretera Tulancingo-Pachuca Km 4.5, Col. Carboneras, Mineral de la Reforma 42184, Hidalgo, Mexico; he214820@uaeh.edu.mx (J.H.-M.); re220359@uaeh.edu.mx (K.A.R.-P.); 2 Área Académica de Enfermería, Instituto de Ciencias de la Salud, Universidad Autónoma del Estado de Hidalgo, Circuito Ex. Hacienda la Concepción, Carretera Pachuca-Actopan, San Agustín Tlaxiaca 42160, Hidalgo, Mexico; abigail_garcia@uaeh.edu.mx; 3 Área Académica de Nutrición, Centro de Investigación Interdisciplinario, Instituto de Ciencias de la Salud, Universidad Autónoma del Estado de Hidalgo, Circuito Ex. Hacienda, La Concepción S/N, Carretera Pachuca-Actopan, San Agustín Tlaxiaca 42160, Hidalgo, Mexico; esther_ramirez@uaeh.edu.mx

**Keywords:** bioactive compounds, extraction methods, functional food, human health, nutritional profile, oxidative stress, sprouts

## Abstract

Due to their outstanding nutritional profile, the consumption of seeds has been an essential source of nutrients. These foods have a unique composition, containing carbohydrates, proteins, lipids, fiber, vitamins, minerals, and bioactive compounds in the same food matrix. Furthermore, the nutritional profile can naturally be maximized and optimized through the germination process through two key methods: degradation of macromolecules and biosynthesis of metabolites, which favors an increase in the concentration of bioactive compounds, such as phenolic compounds. The extraction of these compounds has been studied in various plant fractions, including roots, stems, leaves, fruits, and seeds, using different extraction techniques. Among these, ultrasound-assisted extraction has gained popularity due to its efficiency and yield, considering specific parameters to maximize the bioactive yield. These advances have allowed us to evaluate the potential of the extracted compounds as preventive agents in cardiovascular and degenerative diseases, showing promising results in preventive medicine. Recent studies have shown that cereals possess anti-lipid, anti-hypercholesterolemic, anti-diabetic, anti-inflammatory, and antibiotic properties, mainly due to their antioxidant capacity. This work describes the effects of germination on the nutritional profile, presents benefits to human health through seed consumption, and refers to a collection of strategies to improve the extraction process.

## 1. Introduction

Scientific research has focused on seeds as they are a relevant source of carbohydrates, fiber, proteins, amino acids, enzymes, vitamins, and minerals within the same food matrix [1]. Their nutritional characteristics can be improved through the germination process, a vital period for seeds that includes the activation of metabolism and the anabolism of nutrients necessary for embryo development [2]. Germination eliminates the dormant stage of seeds by activating and releasing endogenous enzymes such as proteases, amylases, and lipases [3]. These enzymes can enhance the breakdown process of complex molecules such as proteins, carbohydrates, and lipids into simple components. Among these, amino acids, simple sugars, and unsaturated fatty acids have been demonstrated to improve the digestibility of grains [1]. Within these metabolic processes, bioactive compounds such as phenolic compounds, amino acids, peptides, oligosaccharides, fiber, free fatty acids, or short-chain fatty acids generate a positive impact on human health mainly due to their antioxidant activity through the consumption of sprouts [4]. New plant growths are products of popular consumption in Europe, Australia, the United States, and Asia, owing to adequate nutrient contents and health-promoting phytochemical compounds [5]. Sprouts grown directly from plant seeds increase the content of bioactive compounds and enhance their bioactivities [6]. For example, sprouts are known to contain more soluble sugar, chlorophyll, carotenoids, and amino acids than seeds. Additionally, germination has been observed to decrease the levels of anti-nutritional components and enhance the digestibility and sensory attributes [7,8].

Bioactive compounds are substances found in various food sources that have a specific purpose in consumer health [9]. A wide variety of foods may contain compounds capable of preventing chronic diseases and inflammation. Furthermore, studies have demonstrated their ability to hinder some types of cancer, arthritis, cardiovascular diseases, or neurodegenerative disorders [10]. These are non-communicable diseases (NCDs) with a prevalence of around 32 million people worldwide with a condition. One of the main causes is an unhealthy diet, including excessive consumption of salt, sugars, and fat [11].

Among bioactive compounds, phenolics have been shown to regulate human health through their antioxidant properties [4]. These compounds have a series of aromatic rings and one or more hydroxyl groups. There are more than 8000 known phenolic structures with various configurations, from which simple chemicals such as phenolic acids to complex substances such as tannins have been described [12]. These compounds are widely distributed within the plant kingdom and have relevant functions for plants, as a defense mechanism against biotic and abiotic threats [13]. On the other hand, they also fulfill relevant functions for the human body by enhancing protection and resistance to oxidative stress, providing antioxidant and anti-inflammatory activity against various chronic diseases [10]. Furthermore, they are described as influencing enzymes and cellular receptors, generating biological functions beyond their antioxidant properties [1]. The structural characteristics of these compounds give them solubility, polarity, and separation capacity. Therefore, they can be extracted from different conventional or non-conventional extraction techniques that use assisted methods, allowing the production of extracts rich in nutrients and bioactive compounds [14]. Temperature, extraction medium (solvents), and time are parameters that have a significant impact on maximizing extraction yields, thus achieving higher [15]. Therefore, this research describes the germination process and the changes in the seed’s nutritional profile that generate bioactive compounds. There are references to the effects of some seed compounds on human health and descriptions of parameters that affect the performance of different methods employed for their extraction.

## 2. Germination Process

**Figure 1:**
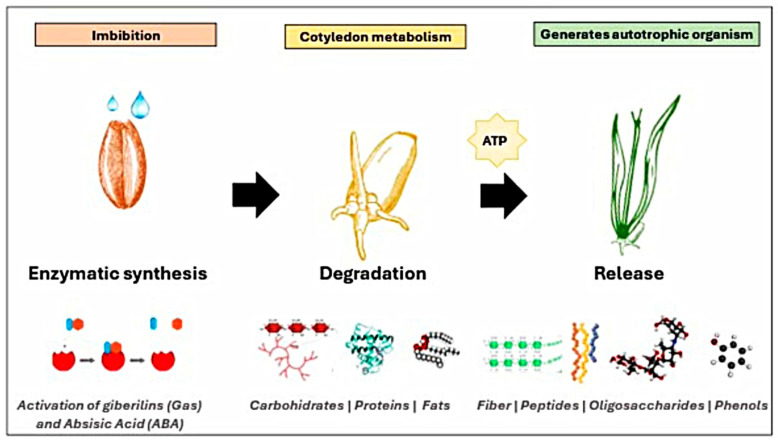
Enzymatic activation in germination.

Germination is a physiological process controlled by the seed embryo, beginning with water absorption (imbibition) and ending when a plant fraction passes through the surrounding layers (emergence), resulting from the elongation of the embryonic axis [16]. Imbibition reactivates the metabolism of nutrients such as carbohydrates, proteins, and fats, producing adenosine triphosphate (ATP), as well as inducing respiratory activity (Figure 1). The synthesis of deoxyribonucleic acid (DNA), ribonucleic acid (RNA), and proteins initiates. Enzymatic activity promotes the degradation of the cotyledons’ reserve material [17]. Through its degradation, phenolic compounds bound to cell wall components such as cellulose and proteins are released [2]. In each of the metabolic processes of the nutrients present in the cotyledons, precursors of bioactive compounds can be released through enzymatic activity [17]. For example, the amino acids used to synthesize phenolic compounds are released through proteolysis. Glucose can be used as an initial precursor of the acetate-malonate pathway to synthesize phenolic compounds [2]. Endosperm degradation promotes macronutrient metabolism, generating free fractions of fiber, peptides, or some oligosaccharides with the potential to prevent human health [18]. In addition, physical changes occur, such as seed coat rupture and organelle and membrane repair [16], to provide the necessary nutrients for the seed’s development into an autotrophic organism. 

On the other hand, the hormones gibberellings (Gas) and abscisic acid (ABA) are considered the main hormones involved in the regulation of development, dormancy, storage, and germination [19]. These hormones stimulate the synthesis of enzymes such as amylases, glucosidases, rate-limiting dextransases, lipoxygenases, and proteases that participate in endosperm degradation to generate bioavailable molecules [20,21]. Furthermore, these hormones will ultimately aid in promoting the concentration of nutrients while reducing antinutritional factors such as phytates, tannins, and oxalates. Following germination, sprouts with a high content of bioactive compounds are obtained [22].

## 3. Macronutrient Metabolism

### 3.1. Carbohydrate Metabolism

**Figure 2:**
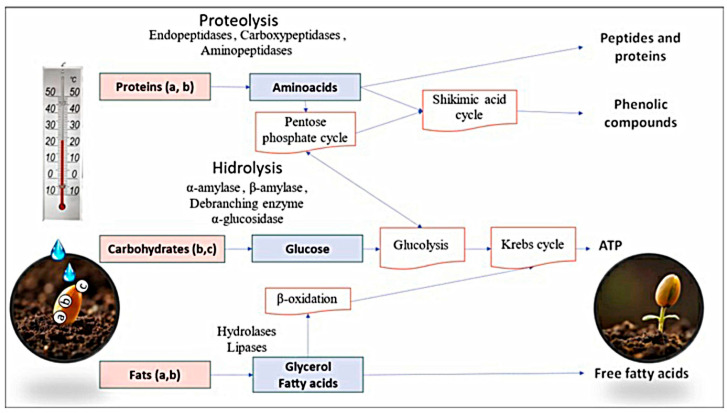
Seed metabolism: (a) corresponds to the seed embryo, (b) refers to the location of the endosperm, and (c) refers to the aleurone layer.

Starch is the primary carbohydrate reserve in seeds. It is stored in the endosperm and hydrolyzed by α-amylase, which participates in breaking the α (1–4) glycosidic bond. Dextrinases intervene in the α (1–6) glycosidic bond of amylose or amylopectin, producing short-chain sugars such as glucose, maltose, and dextrins, respectively. The complete starch digestion is conducted by α-amylase, β-amylase, a debranching enzyme, and α-glucosidase to supply energy to the embryo during cell division. Furthermore, mechanical barriers are removed to allow radicle emergence during germination [16,18]. The energy needed for cellular metabolism is obtained through starch hydrolysis, which generates glucose molecules suitable for glycolysis. Subsequently, the pyruvate molecule enters the Krebs cycle to obtain adenosine triphosphate (ATP) (Figure 2). On the one hand, carbohydrate metabolism releases phenolic compounds bound to cell wall components such as pectins, cellulose, hemicellulose, starches, etc. [12]. Moreover, phenolic compounds can be synthesized during sugar metabolism through the pentose phosphate cycle, where tyrosine, phenylalanine, and tryptophan, precursors of phenolic compounds, are synthesized [2].

### 3.2. Protein Metabolism

The metabolism of storage proteins involves endopeptidases, carboxypeptidases, and aminopeptidases, which interact to hydrolyze the carboxyl and amino terminal, releasing peptides or amino acids [16]. The embryo uses them to develop, and new protein synthesis is relevant for seedling growth [3,18,23]. They are also precursors to phenolic compounds or can be used to synthesize glucose through the pentose phosphate pathway as an energy source (Figure 2). Furthermore, this process alters the composition of amino acids because they are necessary to maximize enzymatic activity [1] or to perform various functions. For example, glutamic acid is a precursor of glutamine, which participates in signaling during germination and root development. In a similar way, arginine participates in nitrogen reassimilation [23], and tyrosine and phenylalanine are involved in the biosynthesis of phenolic compounds. Proteolytic degradation is limited by the formation of stable aggregates, thus ending the proteolysis process during germination [6].

### 3.3. Fat Metabolism

Triacylglycerol is the main fat present in seeds. It is hydrolyzed primarily by triacylglyceride hydrolases and lipases, which release free fatty acids and glycerol [1]. Fatty acids get to the glyoxysomes where they are metabolized by the β-oxidation pathway [23] to produce succinate by coupling with glyoxylate. The synthesized succinate enters the tricarboxylic acid cycle to form malate or oxaloacetate, precursors of sucrose. Finally, they are metabolized as an immediate energy source to support embryo development, providing energy (Figure 2) [24,25]. On the other hand, gluconeogenesis can be activated, which uses glycerol as a substrate to produce glucose essential to synthesize sucrose or structural polysaccharides convenient for the seedling [10]. Later, these can have the same function as the sugars from carbohydrate degradation and synthesize phenolic compounds necessary in the oxidative stress present due to the metabolic activity of the embryo [16].

### 3.4. Metabolism of Phenolic Compounds

Phenolic compounds are secondary metabolites that originate from the primary metabolism of plant cells. They play a crucial role in regulating biological functions and offer various health benefits to humans [9].

During germination, the levels of phenolic compounds increase, which is facilitated by de novo biosynthesis during the metabolism of macromolecules into amino acids, glucose, and acetyl coenzyme A [26]. This process provides substrates for the synthesis of phenolic compounds through various metabolic pathways such as the propionic, oxidative pentose phosphate, hydrolyzable tannin, or acetate-malonate pathways in response to abiotic stress or the scavenging of reactive oxygen species (ROS) [23]. During this period, the expression of enzymes such as phenylalanine ammonia lyase (FAL) and tyrosine ammonia lyase (TAL) increases. These will later intervene in the activation of defence pathways by promoting the biosynthesis of phenolics through the phenylpropanoid metabolic pathways and the shikimate pathway, the latter being the usual route (Figure 2) [13,18,27]. These metabolic pathways alter basic structures by enzymatic reactions of polymerization, methylation, acylation, phosphorylation, hydroxylation, glycosylation, or oxidation, generating a wide variety of compounds [28].

During the phases of germination, phenolic compounds that are covalently bound to cell wall components such as cellulose, pectin, lignin, hemicellulose, proteins, or carbohydrates [29] are converted into free phenolic compounds by endosperm degradation [30]. These processes are promoted as a response to the oxidative (biotic) stress generated by cellular respiration and abiotic stress as a defence mechanism against UV radiation, temperature, or pests [31].

## 4. Modification of the Nutrient Profile During Germination

Germination leads to changes in the nutrient composition of seeds due to metabolic processes that can benefit users’ health. During this process, the glycemic index of seeds decreases as reserve carbohydrates are used as an energy source for embryo development. Additionally, the fat content is reduced, and short-chain fatty acids are released [10]. Furthermore, protein content and antioxidant activity are maximized due to their synthesis derived from sprout development [32,33]. As shown in Table 1, cereals primarily have a higher phenolic compound content, contributing to their disease-preventive properties. In contrast, legumes contain protein as their main nutrient [32]. Bioactive peptides that have disease-preventive properties can be derived from these sources. Among cereals, barley, corn, sorghum, and wheat are the main grains with the highest content of phenolic compounds (Table 1). Legumes such as broad beans, lentils, and kidney beans have a high protein content, which confers preventive activity by releasing peptides or synthesizing phenolic compounds [27]. In general, the total contents of sugars, proteins, ash, phenolic compounds, and fiber increase after the germination process, unlike starch and fat, which tend to decrease.

The germination process has been investigated under various conditions to improve its nutritional composition. For example, during a period of between 1 and 7 days, 20–30 °C temperature ranges and a relative humidity between 85 and 100% were reported [1]. Accordingly, different effects on the change in the nutrient profile have been observed. Medhe et al. (2023) [38] and Yan et al. (2024) [37] mentioned that they obtained the maximum nutrient content 5 days after germination of bean and wheat, respectively. Levent and Aktas (2024) [41] maximized the nutrient profile of lentil sprouts by germinating them for 4 days at 25 °C with 80–90% relative humidity and no light. On the other hand, Levent and Aktas (2024) [41] reported a higher content of total phenolic compounds using barley and compared the results reported by García-Castro et al. (2024) [34]; Islam et al. (2021) [30] observed 84% and 99% less content. In their research with beans, Rizvi et al. (2024) [32] mention 84% more fat content three days after germination, according to what was reported by Şenlik and Alkan (2023) [27], who describe their results four days after germination. This could indicate the use of lipids as an energy source. Further to this, in their evaluation of wheat, Perveen et al. (2024) [18] reported 25% higher ash content, 27% more protein, and 64% higher fat content after 2 days of germination compared to Şenlik and Alkan (2023) [27], who used similar germination conditions.

Seeds contain relevant components such as carbohydrates, proteins, fats, vitamins, amino acids, and fatty acids essential nutrients to human health. Consuming these foods regularly correlates with a reduced risk of chronic diseases [33]. Furthermore, improving their nutritional profile through germination can promote their disease-preventive capacity, primarily due to their antioxidant effects. Information regarding the disease-preventive capacity of seeds is presented below.

## 5. Effect of Bioactive Compounds on Health

Numerous studies have reported that seeds contain bioactive compounds that have demonstrated their ability to prevent the development of diseases (Table 2). Among these are phenolic compounds that exhibit antioxidant effects and free radical scavenging, which confer their ability to reduce the risk of chronic diseases by preventing oxidative damage [42].

Oxidative stress relates to the development of various chronic diseases; it is associated with cell damage by generating cell apoptosis and links to diverse persistent diseases. Under normal conditions, human metabolism generates adequate amounts of reactive oxygen species (ROS) to maintain regular physiological activity [43]. This effect is due to their participation in cellular signal transduction, promoting homeostasis. However, excessive ROS is involved in the pathogenesis of various diseases by attacking cell membranes, proteins, or DNA [44,45]. Therefore, bioactive compounds in seeds, primarily phenolics and peptides, have been studied for their antioxidant capacity.

Chakraborty et al. (2023) [44], in their in vivo study with rice extracts (Table 2), when supplementing the diet of rats, observed a reduction in the levels of superoxide dismutase (SOD), catalase (CAT), glutathione peroxidase (GPx), and reduced glutathione (GSH) in diseased rats, with a decrease of 29.83%, 25.64%, 28.75%, and 34.68%, respectively, compared to rats supplemented with 400 mg/kg of rice extract. These enzymes are considered the first line of defense in maintaining redox balance, thus demonstrating the antioxidant benefits of cereals.

On another note, some unfavorable health effects can be promoted by fat oxidation, causing the accumulation of lipid plaques that can even produce chronic inflammation, an underlying cause of cardiovascular diseases [46]. Free radicals can immediately react with oxygen and cause lipid oxidation, damaging the integrity of the cell membrane [43]. Therefore, regulating the serum levels of these components through β-glucans, unsaturated fatty acids, and phenolic acids has been shown to inhibit adipogenesis and the oxidative damage generated by them [47].

Moreover, in their study, Aly et al. (2024) [48] report a 14.5% reduction in cholesterol after 12 weeks of feeding mice with barley-based bread (Table 2). In a similar way, Bouaziz et al. (2023) [49], in their in vivo study, report a 25–31% decrease in low-density lipoprotein (LDL) cholesterol in rats fed a diet supplemented with 5% barley β-glucans.

According to these reports, the ability of barley seeds to improve health through their antioxidant effects has been described and attributed to their bioactive compound content that increases intracellular antioxidant enzyme activity, eliminates free radicals, and inhibits oxidative stress damage by reducing inflammation [43]. Furthermore, they have demonstrated their ability to regulate fat oxidation by decreasing LDL, thereby reducing free radicals with relevant effects within the inflammatory processes. When these diminish, they provide preventive effects for human health [50].

### 5.1. Regulation of Inflammatory Processes

Inflammatory processes can lead to negative health effects. Research has demonstrated that both phenolic compounds and fiber can help regulate inflammation. This regulation may help prevent the onset of degenerative conditions such as diabetes or epithelial atrophy [51,52]. Inflammation is a complex reaction of the immune system triggered by infections, damaged cells, or irritants [46]. Cyclooxygenase (COX) inhibition is a therapeutic target to address this condition. Cyclooxygenases are generated in two main forms: Cyclooxygenase 1 (COX-1) is found in most tissues. Cyclooxygenase 2 (COX-2) is the response to physical stimuli or pathogens. It is released into tissues to produce prostaglandins, triggering inflammation. The anti-inflammatory properties of seed extracts are due to their ability to interfere with inflammatory signaling, affecting the nuclear factor kappa beta (NFκβ) pathway involved in various biological processes such as inflammation and COX inhibition [53].

Eid et al. (2024) [50], in their in vitro study, showed that barley extracts (Table 2) significantly reduced inflammation by suppressing COX-1 was demonstrated with a half-maximal inhibitory concentration (IC50) of 7.48 μg/mL with methanolic extract. In contrast, COX-2 activity was demonstrated with an IC50 of 3.25 μg/mL. Using bean extracts (Table 2) that contain phenolic compounds, Fonseca Hernandez et al. (2023) [53] inhibited COX-2, decreasing the cellular inflammatory response. Furthermore, in an in vitro study, the authors of [54] described the anti-inflammatory properties of barley leaf extracts, attributed to the presence of saponarin, a natural compound found in leaves that can regulate the production of the cytokine IL-17, controlling inflammatory reactions.

Similarly, fiber, a component normally found in seeds, has been shown to reduce inflammation. For example, Gao et al. (2022) [46] demonstrated the positive effect of oat fiber on the toll-like receptor 4 (TLR 4) signaling pathway, reducing protein expression and decreasing inflammation (Table 2). Naturally, fiber is also essential for its ability to improve the metabolism of the intestinal microbiota, providing additional benefits to the human body.

### 5.2. Regulation of the Intestinal Microbiota and Antibiotic Effects

Fiber has benefits for regulating the intestinal microbiota, which in turn promotes various positive effects on the host’s metabolism, generating metabolites with preventive potential and maintaining the intestinal mucosa in adequate condition [46].

Supplementation with 5% barley β-glucans (Table 2) in the basal diet promoted higher development of lactobacillus in the intestinal tract of rats compared to a high-fat diet. The development of beneficial microorganisms promoted the protection of the intestinal mucosa [49]. In the same vein, the presence of fiber slows the intestinal passage of the food bolus, promotes satiety, and reduces the gastric absorption of glucose and sterols, consequently decreasing LDL in addition to diminishing the glycemic index [48,49].

Regarding pathogenic microorganisms, the seeds exhibit antibacterial and antifungal activity [55]. This capacity is due to phenolic compounds with hydroxyl radicals that cause protein denaturation, altering the integrity of the cell membrane [56].

Abirami et al. (2021) [56] mentioned, in their study, that corn extracts (Table 2) were effective in inhibiting *Aspergillus niger*, *Aspergillus flavus*, and *Aspergillus brasiliensis* at a concentration of 2 mg/20 mL of solution, and attributed this to the presence of tannins, saponins, and flavonoids. Advances in research on bioactive compounds have led to the search for improvements in their extraction. We must consider appropriate conditions such as temperature, pressure, solvent, and time [57]. Below is information for optimizing extraction through the maceration process.

**Table 2:** Health effects promoted by bioactive compounds from cereals.

Seed	Bioactive Compound	Bioactivity	Study In Vivo/In Vitro	Preventive Effect	Reference
Cereals					
Barley	β-glucan	Anti-obesity, anti-glycemic, hypolipidemic and microbiota regulation	in vivo	Decreased fasting blood glucose Sasietogenic effect Increased gut microbiota	[49]
	Phenols	Hepatoprotection	in vivo	Regulation of oxidative stress and inflammation Reduction of proinflammatory factors	[48]
Corn	Polyphenols	Antibiotic and antifungal	in vitro	Protein denaturation	[56]
		Anti-inflammatory	in vitro	Inhibition of inflammatory markers Inhibition of reactive oxygen species (ROS) production	[50]
Rice	Polyphenols	Antihyperlipidemic	in vivo	Inhibition of superoxide dismutase (SOD), catalase (CAT), and glutathione (GPx) Reduction of blood lipids	[44]
		Antidiabetic	in vivo	Beta-cell viability and proliferation Reduction of oxidative stress Insulin regulation	[49]
Oat	β-glucan	Antihypercholesterolemic	in vivo	Depletion of liver cholesterol	[47]
	Fiber	Anti-inflammatory and anti-atherosclerotic	in vivo	NLRP3 inflammasome inhibition Decrease in lipopolysaccharides	[46]
Wheat	Peptide	Antioxidante	in vitro	Reduction of MDA, a byproduct of lipid peroxidation	[45]
	Lunacin	anti carcinogenic	in vitro	Inhibition of histone H3 and H4 acetylation Inactivation of tumor suppressors	[58]
Legumes					
Bean	Polyphenols	Anti-inflammatory	in vitro	COX-2 inhibition	[51]
		Antidiabetic	In vitro	Increased glucose uptake	[59]
Faba	Peptide	Antioxidant	in vitro	Protection against oxidative damage	[60]
	Polyphenols	Antioxidant	in vitro	Protection of DNA against oxidative damage	[61]
Lentil	Polyphenols	Anti-inflammatory	in vitro	Inhibition of COX-2 and nitric oxide (NO)	[62]

## 6. Extraction of Phenolic Compounds

The extraction of phenolic compounds from a food matrix is the first step in evaluating their antioxidant properties and their preventive capacity in various conditions related to oxidative stress [14]. Their extraction has been assessed using various methods that offer advantages and disadvantages depending on the extraction objective [15]. For this, we must consider the complexity of the phenolic compound since its structure influences the extraction yield through interaction with solvents. In addition, we must pay attention to the food source where they are present to choose the appropriate conditioning [42]. It is relevant to note that there is no universal method to recover phenolic compounds or any specific group. Therefore, several factors must be considered: the type of sample, the target compounds, the purpose of the análisis, and the availability of the technique [15]. Please note that sample preparation is a relevant step before the extraction technique, which is normally performed through operations such as drying, homogenization, filtration, grinding, or hydrolysis to facilitate the release of compounds. Typically, researchers utilize solvent extraction; however, ultrasound- and microwave-assisted techniques have become popular because they are faster and allow more efficient extraction [63].

### 6.1. Use of Solvents

Phenolic compounds are generally extracted using solvents such as ethanol, propanol, methanol, water, chloroform, ethyl acetate, acetone, or ether. These differ in their physicochemical characteristics, primarily in their polarity, which influences the compounds directly [14]. For this reason, the use of solvent mixtures is commonly mentioned to ensure the desired objective. According to the reported literature, the conclusion is that there is no ideal solvent because its efficiency depends on the nature of the target compound, its degree of polymerization, its polarity concerning the solvent, as well as the food source of the compound [64]. Furthermore, it is relevant to consider the process parameters used in the extraction; the characteristics of the solvents, such as boiling point, reactivity, compatibility with other solvents, and viscosity, will also depend on this [14]. It is essential to conduct experimental runs before the final process to select the solvent that demonstrates the best results for achieving the desired objective.

### 6.2. Extraction Techniques

#### 6.2.1. Conventional Techniques

Extraction is conducted through conventional processes, such as mechanical agitation, maceration, decoction, infusion, and the Soxhlet method, techniques commonly used due to their ease of application [65,66]. Other procedures, such as filtration, decantation, or clarification, can accompany these techniques to fractionate some compounds [14]. However, their application has begun to be replaced by emerging techniques.

#### 6.2.2. Emerging Techniques

**Figure 3:**
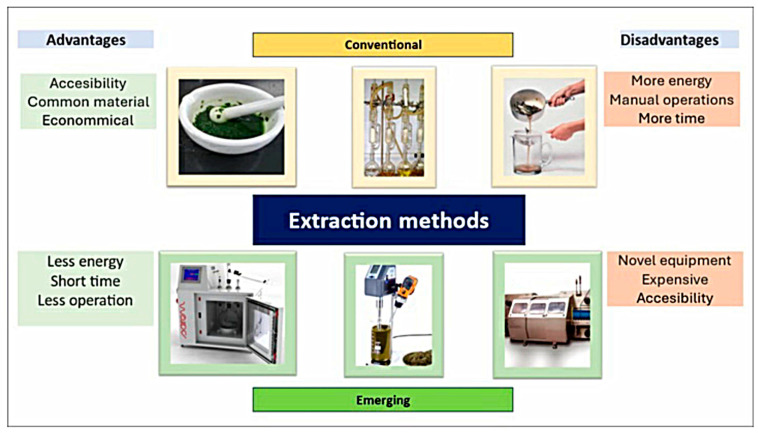
Extraction methods of phenolic compounds.

Emerging techniques, such as ultrasound-assisted extraction using an ultrasound bath or probe, microwaves, high pressures, enzymatic treatments, or supercritical fluids, to name a few [23], are recognized for requiring low volumes of solvents, time, and energy (Figure 3). This characteristic is due to their ability to guarantee cell lysis, enabling extraction efficiency. On the other hand, hybrid techniques are currently being integrated, combining emerging techniques that have resulted in improved efficiency [66]. Conventional extraction techniques or those assisted by microwaves and ultrasound are commonly used due to their lower cost, accessibility, and availability [12]. In another order of ideas, the efficiency of different extraction methods will ultimately depend on numerous factors, such as the bioactive compound, the food matrix that contains it, the pretreatment of the sample, and the extraction medium [12]. Due to these factors, it is necessary to previously adapt the solvent together with the technique that best suits the desired objective [14].

### 6.3. Strategies to Optimize the Extraction Process

Various investigations have involved performing extractions using conventional techniques, as shown in Table 3. However, ultrasound-assisted, microwave-assisted, and high-pressure techniques are beginning to gain relevance due to their higher yields.

As seen in Table 3, emerging techniques are characterized by shorter operating times, maximizing the content of phenolic compounds. High-pressure assistance has emerged as an alternative to conventional processes by generating a rupture effect on the cell wall, allowing the release of phenolics [67]. Using ultrasound assistance in rice extraction yields a 2.72 times higher phenolic content. Furthermore, when using methanol as a solvent, the extraction temperature also influences these results, as it facilitates the desorption and solubility of molecules within the solvent [14]. Temperature plays a crucial role in the extraction process, as it positively influences the effectiveness of the operation. Despite this, it is essential to adjust the extraction temperature because phenolic compounds are unstable at temperatures above 80 °C [68,69].

According to the aforementioned, ultrasound assistance has had a strong impact when using low extraction temperatures. Within this subject, authors [70] have recommended ultrasound assistance with low frequencies (37 KHz) and temperatures no higher than 35 °C to maximize extraction. For example, [63] obtained, through ultrasound assistance in barley extracts (Table 3), 6.59 and 2.54 times higher phenolic content compared to García-Castro et al. (2024) [30] and Eid et al. (2024) [44]. This may be because ultrasound treatment generates a cavitation effect that raises pressure, increases membrane permeability, and promotes rupture of the cell wall, allowing water entry. This effect increases the phenol content by activating enzymatic metabolism for its synthesis [13]. Furthermore, molecular motion is accelerated, which induces physical alteration of plant tissues [57]. Mass and energy transfer is facilitated, increasing extraction yield [67] due to a slight indirect temperature increase in the solution, influencing the solubility of the compounds [57].

In another order of ideas, the maceration process can enhance the phenolic compounds’ solubility and mass transfer. García-Castro et al. (2024) [34] mention that maceration influences the concentration of phenolic compounds by activating hydrolytic enzymes that allow their release into the medium by solubilizing them in water in a temperature range of 45–78 °C, allowing for the maximization of their content by up to three times. Exemplifying this (Table 3), Yan et al. (2024) [37] obtained a 1.78 times higher content of wheat phenols than Şenlik & Alkan (2023) [27], using a mostly dissolved solution. In the same way, a higher yield was observed by Contreras et al. (2024) [71], who used a 60% higher solution than Yu et al. (2024) [13], obtaining a 2.15 times higher phenol content. Water is a relevant parameter in the extraction process. As mentioned, this effect is attributed to the ability of water to interact with carboxylic and hydroxyl compounds due to its high polarity and its characteristics as a solvent by forming hydrogen bonds [15,72].

Another relevant parameter to consider in the extraction process is the compounds found free and bound to other macromolecules. This effect is observed through germination or milling of the grain, processes that can release compounds bound to macromolecules such as pectin, lignin, cellulose, proteins, or carbohydrates, facilitating their extraction with solvents [29]. According to this, the results obtained by Eid et al. (2024) [50], when using the same solvent, report an increase of 2.59 times the phenol content compared to that reported by García-Castro et al. (2024) [34] and Xia et al. (2022) [73], probably due to the presence of free phenols generated by a more exhaustive milling process. In support of the foregoing, [24] clarify that, with these processes, it is possible to release enzymes present in the seeds, which intervene in the synthesis of bioactive compounds by providing them with the ideal conditions in the extraction process, for which the presence of water plays a relevant role as a catalyst. Finally, Yan et al. (2024) [37] demonstrated the importance of stress induced on the seeds and maximized the content of phenolic compounds. In the study, the authors obtained better results by stressing the seed during the germination process with acidified water (pH = 5), allowing them to observe a 3-fold increase in the phenolic content.

**Table 3:** Phenolic compounds extraction.

Seed	Extraction Method	Parameters			Phenols (mg GAE/100 g)	Reference
		Solvent	Time (min)	Temperature (°C)		
Cereals						
Barley	MT	Water	34	-	57	[48]
			90	70	22	[34]
	US	Water	10	-	145	[63]
Corn	MT	Methanol 80%	120	-	96	[27]
	US		4	25	349	[74]
Oat	US	Methanol 80%	30	-	30	[75]
Rice	HP	Ethanol	8	97	210	[67]
	US	Water	25	-	77	[76]
Sorghum	US	Methanol 80%	30	25	2667	[77]
Wheat	MT	Methanol 80%	120	-	47	[27]
		Ethanol 70%	120	60	84	[39]
	US	Hexane	10	-	40	[78]
Legumes						
Bean	MT	Methanol 80%	120	-	145	[27]
	US	Ethanol 80%	120	45	763	[13]
		Ethanol 20%	60	40	1648	[71]
		Ethanol 80%	49	-	610	[79]
Faba	MT	Methanol 70%	120	-	269	[23]
	US	Ethanol 80%	45	25	474	[80]
Lentil	MT	Methanol 80%	120	-	76	[27]
	MW	Ethanol 25%	5	-	68	[81]
Soy	MT	Methanol	60	-	24	[82]
Pea	MT	Water	1440	30	1100	[83]
	US		30	30	1110	

(MT) maceration; (US) ultrasound; (HP) high pressure; (MW) microwave; (min)—minutes; (°C) Celsius; mg GAE/100 g—milligrams of gallic acid equivalents per 100 g of sample.

## 7. Conclusions

Germination primarily potentiates the phenolic profile of the seed due to its synthesis and release from induced stress during this process. Fiber and protein are improved to a lesser extent. Some compounds, such as sugars and fats, decrease. These changes depend on the seeds and varieties’ metabolism. Since these nutrients are enhanced, they can prevent adverse human health consequences, as these compounds have proven their antioxidant, anti-inflammatory, antihyperlipidemic, anticarcinogenic, antibiotic, and microbiota regulating properties, among others; this is mainly due to their reducing capacity. Seeds and sprouts have been the subject of various research studies, providing scientific support for their use as functional food. However, further research is required on the effects of bioactive compounds on the metabolic mechanisms involved in human health. For this reason, the extraction of phenolic compounds has been studied primarily through various methods, both conventional and emerging techniques. For improved yields, it is recommended to consider an extraction temperature below 80 °C, apply appropriate water–solvent ratios, pre-condition the sample, and explore emerging techniques. Additionally, more information is required regarding the bioavailability and toxicity of phenolic compounds, and an in-depth review of the changes in the nutritional profile of various seeds during germination, which represents a crucial area of research to enhance the benefits that sprouts provide to human health.

## Figures and Tables

**Table 1:** Modification of the nutritional profile of seeds through germination.

Seed	Germination Conditions			Change in Nutritional Profile		
	Time (days)	Temperature (°C)	Relative Humidity (%)	Nutrient	Increase/Decrease (%)	Reference
Cereals						
Barley	7	24	70	Ferulic acid *	384	[34]
				Syringic acid *	21	
				Total sugars *	140	
				Total flavonoids ****	316	
				Protein **	106	
	4	25	75	Ash **	6	[30]
				Starch **	−8	
				Fat **	4	
		24	-	Total phenols ***	27	[27]
				Soluble fiber **	1	
Corn	4	24	-	Total Sugars *	−3	[27]
				Ash **	−3	
				Total Phenols ***	80	
				Soluble Fiber **	6	
				Fat **	−38	
				Protein **	42	
Millet	2	30	-	Total phenols ***	31	[35]
				Total flavonoids ****	−17	
Oat	2	35	95	Total phenols ***	165	[36]
Rice	1	35	95	α-Tocopherol *	43	[25]
				Ferulic acid *	442	
				GABA *	1169	
				Kaempferol *	4	
				Quercetin *	30	
Rye	2	25	95	Total phenols ***	105	[36]
Sorghum	2	30	-	Total phenols ***	−23	[35]
Triticale	2	25	95	Total phenols ***	155	[36]
Wheat	4	24	-	Total Sugars *	−9	[29]
				Soluble Fiber **	8	
	3	25	-	Insoluble Fiber **	−17	[17]
				Ash **	175	
				Gluten **	56	
				Fat **	−23	
				Protein **	21	
	5		60	Total Phenols ***	138	[37]
Legumes						
Bean	5	-	-	Total Sugars *	−3	[38]
				Insoluble Fiber **	−1	
	4	24		Ash **	14	[27]
				Protein **	18	
	2	37	90	Total Phenols ***	169	[13]
	3	25	80	Total Flavonoids ****	36	[32]
				Soluble Fiber **	21	
				Fat **	−30	
Chickpea	5	-	-	Total Sugars *	−11	[29]
				Ash **	−16	
				Total Phenols ***	84	
				Total Flavonoids ****	40	
				Kaempferol *	87	
				Fat **	−13	
Faba	2	30	90	Starch **	−23	[23]
				Total Sugars *	2	
				Ash **	9	
	5	30	-	Total Phenols ***	−68	[39]
	3	23	-	Soluble Fiber **	−12	[40]
				Fat **	−19	
				Protein **	−55	
Lentil	4	24	-	Total Sugars *	−19	[29]
				Total Phenols ***	72	
				Soluble Fiber **	4	
		25	90	Ash **	10	[41]
				Fat **	221	
				Protein **	7	

Measure units * (mg/g); ** (%); *** (mg GAE/g); **** (mg QE/g); (°C)—Celsius; (%) percentage; (mg GAE/g)—milligrams of gallic acid equivalents per gram of sample; (mg QE/g)—milligrams of quercetin equivalents per gram of sample; (mg RE/g)—milligrams of retinol equivalents per gram of sample.

## Abbreviations

ABA	abscisic acid
ATP	adenosine triphosphate
CAT	catalase
COX	cyclooxygenase
DNA	desoxyribonucleic acid
Gas	gibberellings
GPx	glutathione peroxidase
GSH	reduced glutathione
KHz	kilohertz
LDL	low-density lipoprotein
NCDs	non-communicable diseases
NFκβ	nuclear factor kappa beta
pH	Hydrogen potential
RNA	ribonucleic acid
ROS	reactive oxygen species
SOD	superoxide dismutase
TLR 4	toll-like receptor 4
