# Correction: Hernández-Miranda et al. Impacts of Phenolic Compounds and Their Benefits on Human Health: Germination. *Metabolites* 2025, *15*, 425

**DOI:** 10.3390/metabo16020123

**Published:** 2026-02-11

**Authors:** Jonathan Hernández-Miranda, Karen Argelia Reyes-Portillo, Abigail García-Castro, Esther Ramírez-Moreno, Alma Delia Román-Gutiérrez

**Affiliations:** 1 Área Académica de Química, Instituto de Ciencias Básicas e Ingeniería, Universidad Autónoma del Estado de Hidalgo, Carretera Tulancingo-Pachuca Km 4.5, Col. Carboneras, Mineral de la Reforma 42184, Hidalgo, Mexico; he214820@uaeh.edu.mx (J.H.-M.); re220359@uaeh.edu.mx (K.A.R.-P.); 2 Área Académica de Enfermería, Instituto de Ciencias de la Salud, Universidad Autónoma del Estado de Hidalgo, Circuito Ex. Hacienda la Concepción, Carretera Pachuca-Actopan, San Agustín Tlaxiaca 42160, Hidalgo, Mexico; abigail_garcia@uaeh.edu.mx; 3 Área Académica de Nutrición, Centro de Investigación Interdisciplinario, Instituto de Ciencias de la Salud, Universidad Autónoma del Estado de Hidalgo, Circuito Ex. Hacienda, La Concepción S/N, Carretera Pachuca-Actopan, San Agustín Tlaxiaca 42160, Hidalgo, Mexico; esther_ramirez@uaeh.edu.mx

The authors would like to make the following correction to their published paper [1]. The change is as follows:

Reference [19] in the original publication is incorrectly cited and needs to be replaced with the following reference:

19. Tuan, P.A.; Kumar, R.; Rehal, P.K.; Toora, P.K.; Ayele, B.T. Molecular Mechanisms Underlying Abscisic Acid/Gibberellin Balance in the Control of Seed Dormancy and Germination in Cereals. *Front. Plant Sci*. **2018**, *9*, 668. https://doi.org/10.3389/fpls.2018.00668.

The authors state that the scientific conclusions are unaffected. This correction was approved by the Academic Editor. The original publication has also been updated.
